# Fas-Associated Factor 1 Negatively Regulates the Antiviral Immune Response by Inhibiting Translocation of Interferon Regulatory Factor 3 to the Nucleus

**DOI:** 10.1128/MCB.00744-15

**Published:** 2016-03-18

**Authors:** Soonhwa Song, Jae-Jin Lee, Hee-Jung Kim, Jeong Yoon Lee, Jun Chang, Kong-Joo Lee

**Affiliations:** Graduate School of Pharmaceutical Sciences, College of Pharmacy, Ewha Womans University, Seoul, South Korea

## Abstract

This study is designed to examine the cellular functions of human Fas-associated factor 1 (FAF1) containing multiple ubiquitin-related domains. Microarray analyses revealed that interferon-stimulated genes related to the antiviral response are significantly increased in FAF1-knockdown HeLa cells. Silencing FAF1 enhanced the poly(I·C)- and respiratory syncytial virus (RSV)-induced production of type I interferons (IFNs), the target genes of interferon regulator factor 3 (IRF3). IRF3 is a key transcription factor in IFN-β signaling responsible for the host innate immune response. This study also found that FAF1 and IRF3 physically associate with IPO5/importin-β3 and that overexpression of FAF1 reduces the interaction between IRF3 and IPO5/importin-β3. These findings suggest that FAF1 negatively regulates IRF3-mediated IFN-β production and the antiviral innate immune response by regulating nuclear translocation of IRF3. We conclude that FAF1 plays a novel role in negatively regulating virus-induced IFN-β production and the antiviral response by inhibiting the translocation of active, phosphorylated IRF3 from the cytosol to the nucleus.

## INTRODUCTION

The innate immune system, in contrast to the adaptive immune response present only in immune cells, is present in all cells and plays key roles in the host defense against viral infections by sensing and immediately responding to the invading pathogens (1, 2). Intracellular pattern recognition receptors (PRRs), including Toll-like receptors (TLRs), retinoic acid-inducible gene I (RIG-I)-like receptors (RLRs), and nucleotide-binding oligomerization domain containing (NOD)-like receptors (NLRs), recognize pathogen-associated molecular patterns (PAMPs) and activate innate immune signaling pathways, leading to the production of type I interferons (IFN-α/β) and other cytokines. Type I IFNs play a crucial role in limiting viral replication and priming the adaptive immune response (3, 4). IFN-β can be produced in most cell types, and when the cells are infected with a virus, IFN-β expression rapidly increases due to the activation of transcription factors (5). Transcription factor complexes, including interferon regulatory factor 3 (IRF3), nuclear factor kappa B (NF-κB), and AP1, are bound to the regulatory domains of the IFN-β promoter and cooperatively regulate the transcription of IFN-β (6). IFN-β secreted from infected cells binds to type I IFN receptors 1 and 2 (IFNAR1/2) on adjacent cells and then activates the JAK/STAT signaling pathway, which results in the expression of interferon-stimulated genes (ISGs). Some ISGs, such as Mx1, OAS1, and IFIT1, directly interfere with viral replication, while others, including RIG-I, MDA5, and IRF7, indirectly do so by enhancing IFN-β production (7).

The transcription factor IRF3 plays the most critical role in the regulation of virus-induced IFN-β activation. IRF3 is constitutively expressed and localized in the cytoplasm in a latent form. Single-stranded or double-stranded viral RNAs accumulated inside cells after infection are recognized by RLRs and TLR3, which recruit the adaptor proteins mitochondrial antiviral signaling protein (MAVS) and TRIF, respectively (8, 9). These adaptor proteins, MAVS and TRIF, recruit the kinases TBK1 and IκB kinase ε (IKKε), which activate IRF3 by phosphorylating the C-terminal region of IRF3 at seven Ser/Thr residues (Ser385, -386, -396, -398, -402, and -405 and Thr404). Phosphorylated IRF3 forms dimers which shuttle into the nucleus, where they interact with the coactivator CBP/p300 and initiate transcription of target genes, including IFN-β (10, 11). It has been reported that phosphorylation of IRF3 at Ser386 induces dimerization and interaction with CBP (11) and that phosphorylation at Ser396 occurs in response to viral infections (10). Mutation studies confirmed that phosphorylations at Ser386 and Ser396 are important for IRF3 activation and interaction with CBP (12).

The production of IFN-β is essential for protecting cells from virus infection, and aberrant activation of IFN-β production can trigger diseases, such as multiple sclerosis and systemic lupus erythematosus (SLE) (13, 14). Therefore, IFN-β production needs to be tightly regulated. Several positive and negative regulators have been identified. Studies of mechanisms in IRF3 activation as well as in the negative regulation of transcriptional activity of IRF3 are still ongoing. The two negative-regulatory mechanisms so far identified, as already noted, are degradation of IRF3 following its phosphorylation by the ubiquitin proteasome system and posttranslational modifications of IRF3, which inhibit its activity. RAUL, a major ubiquitin E3 ligase, ubiquitinates IRF3 regardless of its phosphorylation status (15), while the E3 ubiquitin ligase RBCK1 and cytoplasmic peptidyl-prolyl-isomerase Pin1 ubiquitinate only phosphorylated IRF3 and trigger its degradation (16, 17). The second negative-regulation mechanism reported to change IRF3 activity is posttranslational modification of IRF3. Protein phosphatase 2A (PP2A) and mitogen-activated protein kinase (MAPK) phosphatase 5 (MKP5) are known to dephosphorylate IRF3 and decrease the IFN response (18, 19). SUMOylations of IRF3 are another known mechanism to decrease IRF3 activity (20).

Thus, phosphorylation is an indispensable step for IRF3 activation, and phosphorylated IRF3 is translocated into the nucleus to bind the IFN-β promoter. However, the mechanism underlying the translocation process remains elusive. Previous studies demonstrated that IRF3 has an active nuclear localization signal (NLS) which is recognized by importin-α receptors and transported to the nucleus (21, 22). IRF3 also has an active nuclear export signal (NES); it is exported from the nucleus via the CRM1-mediated pathway to localize mainly in the cytoplasm in unstimulated cells (23). Following infection, IRF3 resides in the nucleus and interacts with CBP (21, 24). This study reports that FAF1 as a negative regulator of virus triggered the IFN-β signaling pathway by inhibiting the nuclear translocation of phosphorylated IRF3.

Fas-associated factor 1 (FAF1) was first identified as a component of the apoptosis signaling pathway (25). FAF1 is a ubiquitin receptor containing multiple ubiquitin-related domains that include a ubiquitin-associated (UBA) domain and three domains with ubiquitin-like folds, UBL1, UBL2, and ubiquitin-regulatory X (UBX) (26). The N-terminal UBA domain (47 amino acids long) recruits Lys^48^-linkage polyubiquitinated proteins required for FAF1-mediated apoptosis and the stress response (26, 27). The UBL1 domain binds to Hsp70 and regulates its chaperone activity by promoting Hsp70 degradation (28, 29). The C-terminal UBX domain interacts only with valosin-containing protein (VCP; AAA ATPase p97) complexed with the Npl4-Ufd1 heterodimer. This interaction regulates the binding of the polyubiquitinated proteins via the N-terminal UBA domain. FAF1 promotes the degradation of the endoplasmic reticulum-associated degradation (ERAD) substrate in a VCP-Npl4-Ufd1-dependent manner (26, 30). To further understand the cellular functions of FAF1, we investigated the target genes of FAF1 using microarray analysis. This microarray result reveals that FAF1 is involved in negative regulation of a virus-triggered IFN-β signaling pathway, in a novel manner by inhibiting the nuclear translocation of phosphorylated IRF3 and subsequently IFN-β production and thereby inhibiting the cellular antiviral response.

## MATERIALS AND METHODS

### Reagents and plasmids.

The following are the sources of antibodies used in this study: mouse monoclonal Flag antibody was from Sigma (St. Louis, MO); rabbit anti-IRF3, mouse anti-MAVS, mouse antitubulin, rabbit anti-histone deacetylase 1 (anti-HDAC1), and mouse antiactin antibodies were from Santa Cruz Biotechnology (Santa Cruz, CA); rabbit anti-Mx1 and rabbit anti-phospho-IRF3 (Ser386) were from Abcam (Cambridge, United Kingdom); rabbit anti-FAF1 and rabbit anti-glyceraldehyde-3-phosphate dehydrogenase (anti-GAPDH) were from AbFrontier (Seoul, South Korea); rabbit antibodies specific for IRF3, phospho-IRF3 (Ser396), TBK1, TRIF, ISG15, and STAT1 were from Cell Signaling Technology (MA); mouse anti-green fluorescent protein (anti-GFP) antibody was from Life Technologies (CA); mouse anti-RIG-I antibody was from Adipogen AG (CA); mouse antibodies specific for IPO5 and FAF1 were from Abnova (CA). Cycloheximide (CHX; C7698) and leptomycin B (LMB; L2913) were purchased from Sigma (St. Louis, MO).

pISRE-Luc was provided by Greg Barton (University of California—Berkeley, Berkeley, CA). pIFNB-GL3 and pIFNA4-GL3 were provided by John Hiscott (Vaccine and Gene Therapy Institute of Florida, USA), and pCMV-beta-galactosidase (pCMV-beta-Gal, where CMV is cytomegalovirus) was from Eunsuk Hwang (Ewha Womans University, South Korea). The expression plasmids for TRIF and GFP-tagged wild-type IRF3 (IRF3-WT-GFP) and IRF3 with five Ser/Thr residues replaced with ASP (IRF3-5D-GFP) were kindly provided by Joo Young Lee (Catholic University, South Korea) with kind permission from Katherine A. Fitzgerald (University of Massachusetts Medical School, MA) and John Hiscott (Vaccine and Gene Therapy Institute of Florida, FL). Flag-RIG-I N was from Takashi Fujita (Tokyo, Japan). Flag-MAVS and Flag-TBK1 were from Glen N. Barber (University of Miami Miller School of Medicine, FL). Flag-IKKε was from Ki-Sun Kwon (KRIBB, South Korea). pFlag-CMV-2-FAF1 WT, pFlag-CMV-2-FAF1(82–650), pFlag-CMV-2-FAF1ΔUBX (deletion of amino acids [aa] 569 to 650), pFlag-CMV-2-FAF1ΔUBL1-2 (deletion of aa 100 to 270), pFlag-CMV-2-FAF1ΔUAS (deletion of aa 352 to 487), and pFlag-CMV-2-FAF1(1–201) were prepared as previously described (26, 27). pFlag-CMV-2-FAF1(1–351) was generated by cloning. All plasmid constructs were verified by DNA sequencing.

### Cell culture and transfection.

HeLa cells were purchased from the ATCC and cultured in Eagle's minimal essential medium (EMEM) supplemented with 10% fetal bovine serum, 100 units/ml of penicillin G, and 100 μg/mM streptomycin at 37°C in a 5% CO_2_-containing humidified incubator. HEK293T cells and Raw264.7 cells were cultured in Dulbecco's modified Eagle's medium (DMEM) supplemented under the same conditions. HEp-2 cells were cultured in minimal essential medium (MEM) supplemented under the same conditions. For transient overexpression of specific proteins, cells were transfected using LT-1 reagent and analyzed at 24 h or 48 h posttransfection. For gene silencing, FAF1 small interfering RNAs (siRNAs) were obtained from Dharmacon (ON-TARGETplus SMARTpool siRNA; Dharmacon, IL) and Bioneer (Daejeon, South Korea). FAF1 siRNA 1 was from Dharmacon (L-009106-00-0005) and FAF1 siRNA 2 (catalog number 1049605) and a control siRNA were from Bioneer. siRNA 2 targets the consensus sequence in both human and mouse FAF1 (hFAF1 and mFAF1, respectively) proteins. Cells were transfected with siRNAs using DharmaFECT1 according to the manufacturer's protocol at a final concentration of 100 nM.

### Microarray analysis.

HeLa cells were transfected with an FAF1 or control siRNA and collected at 48 h posttransfection. Total RNA was extracted using TRIzol reagent (Invitrogen Life Technologies, CA) and purified using RNeasy columns (Qiagen, CA) according to the manufacturer's protocol. RNA purity and integrity were evaluated by denaturing gel electrophoresis and the ratio of the optical densities at 260 and 280 nm (OD_260/280_) analyzed with a 2100 Bioanalyzer (Agilent Technologies, CA). Total RNA was amplified and purified using an Ambion Illumina RNA amplification kit (Ambion, CA) according to the manufacturer's instructions to yield biotinylated complementary RNA (cRNA). Briefly, 550 ng of total RNA was reverse transcribed to cDNA using a T7 oligo(dT) primer. Second-strand cDNA was synthesized, *in vitro* transcribed, and labeled with biotin-nucleoside triphosphate (NTP). After purification, the cDNA was quantified using an ND-1000 Spectrophotometer (NanoDrop, Wilmington, DE). A total of 750 ng of labeled cDNA samples was hybridized to each HumanHT-12, version 4, expression bead array for 16 to 18 h at 58°C, according to the manufacturer's instructions (Illumina, Inc., San Diego, CA). Array signals were detected using Amersham Fluorolink streptavidin-Cy3 (GE Healthcare Bio-Sciences, Little Chalfont, United Kingdom). Arrays were scanned with an Illumina BeadArray Reader confocal scanner according to the manufacturer's instructions. The quality of hybridization and overall chip performance were monitored by visual inspection of both internal quality control checks and the raw scanned data. Raw data were extracted using the software provided by the manufacturer (Illumina GenomeStudio, version 2011.1; Gene Expression Module, version 1.9.0). Probe signal values were transformed by logarithm and normalized by the quantile method. Statistical significance of the expression data was determined using a local-pooled-error (LPE) test and fold change in which the null hypothesis was that no difference exists among two groups. The false discovery rate (FDR) was controlled by adjusting *P* values using the Benjamini-Hochberg algorithm.

### Luc reporter assay.

The cells were transfected with reporter genes and pCMV-beta-Gal and then, after 24 h, treated with poly(I·C) (10 μg/ml) by transfection. Transfected cells were harvested, and luciferase (Luc) activity and beta-galactosidase activity were measured using a luciferase assay system (Promega, WI) and a Galacto-Light Plus system (Applied Biosystems, CA), respectively, on a luminometer (Luminoskan TL plus, ThermoFisher Scientific, MA). Each experiment was repeated in triplicate, and firefly luciferase activities were normalized to beta-galactosidase activities.

### Reverse transcription-quantitative PCR (RT-qPCR).

Cells were harvested at 48 h posttransfection. Total RNA from these cells was isolated using an RNeasy minikit (Qiagen, CA) and then reverse transcribed using SuperScript II RT (Invitrogen Life Technologies, CA) according to the manufacturer's protocol. Synthesized cDNA was subjected to real-time PCR (AB7300; Applied Biosystems, CA) for amplification in triplicate. PCRs were performed using SYBR green qPCR master mix (Applied Biosystems, CA) and the following specific primers: for hIFN-β (31), 5′-CAA CAA GTG TCT CCT CCA AAT-3′ (sense) and 5′-TCT CCT CAG GGA TGT CAA AG-3′ (antisense); mIFN-β, 5′-CAT CAA CTA TAA GCA CCA-3′(sense) and 5′-TTC AAG TGG AGA GCA CTT GAG-3′ (antisense); hFAF1, 5′-ATT GGG ACT TAG TGG CAG CT-3′ (sense) and 5′-GCA TTA CAG GTC GAA ACG CT-3′ (antisense); hIFIT1, 5′-CCT CCT TGG GTT CGT CTA CA-3′ (sense) and 5′-GGC TGA TAT CTG GGT GCC TA-3′ (antisense); hIFIH1, 5′-TGG TCT CGT CAC CAA TGA AA-3′ (sense) and 5′-CTC CTG AAC CAC TGT GAG CA-3′ (antisense); hGAPDH, 5′-AAG GTC ATC CCT GAG CTG AA-3′ (sense) and 5′-TGC TGT AGC CAA ATT CGT TG-3′ (antisense); mGAPDH, 5′-AGA ACA TCA TCC CTG CAT CC-3′ (sense) and 5′-CAC ATT GGG GGT AGG AAC AC-3′ (antisense). Relative mRNA expression was calculated according to the comparative threshold cycle (*C_T_*) method (ΔΔ*C_T_*), and the GAPDH gene was used as an endogenous control gene.

### Native PAGE.

HEK293T cells were lysed in buffer containing 50 mM Tris-HCl, pH 7.4, 150 mM, NaCl, 1 mM EDTA, 1% NP-40, 5 mM Na_3_VO_4_, 5 mM NaF, and protease inhibitor cocktail (Sigma, St. Louis, MO). Native gels (Bio-Rad, CA) were prerun with 25 mM Tris-HCl and 192 mM glycine, pH 8.3, with 0.4% deoxycholate (DOC) in the cathode chamber for 30 min at 40 mA on ice before samples were loaded. Samples in native buffer (10 μg of protein, 62.5 mM Tris-HCl, pH 6.8, 25% glycerol, and 1% DOC) were loaded, and native gels were run at 15 mA for 60 min on ice. Gels were soaked in SDS running buffer for 30 min, transferred to polyvinylidene difluoride (PVDF) membranes, and then analyzed by Western blotting.

### Immunoprecipitation.

Cells were lysed in lysis buffer containing protease inhibitors [150 mM NaCl, pH 7.4, 50 mM Tris-HCl, 1 mM EDTA, 1 mM phenylmethylsulfonyl fluoride (PMSF), 5 μg/ml aprotinin, 10 μg/ml leupeptin, 10 μg/ml pepstatin A, 5 mM Na_3_VO_4_, 5 mM NaF, 10 mM sodium butyrate, and 1% CHAPS (3-[(3-cholamidopropyl)-dimethylammonio]-1-propanesulfonate)] for 30 min on ice, followed by centrifugation at 4,000 rpm for 15 min. The supernatant was incubated with anti-Flag antibody or anti-GFP antibody for 2 h at 4°C, and the lysate-antibody complexes were incubated with protein G-Sepharose 4 Fast Flow beads for another 1 h at 4°C. The precipitated beads were washed six times with lysis buffer to remove nonspecific binding. The immune complex was eluted with gel sample buffer, separated by SDS-PAGE, and analyzed by Western analysis.

### Cellular fractionation.

HeLa cells (2 × 10^6^) were lysed in hypotonic solution (10 mM HEPES, pH 7.9, 1.5 mM MgCl_2_, 10 mM KCl, 1 mM EDTA, 5 mM Na_3_VO_4_, 5 mM NaF) containing protease inhibitor cocktail (Sigma, St. Louis, MO) for 30 min at 4°C to swell the cells. Cell lysates were centrifuged at 4,000 rpm for 25 min at 4°C. The pellet was washed and solubilized with 150 μl of gel sample buffer and then used as the nuclear fraction. The supernatant was immediately subjected to Western blot analysis as the cytosolic fraction.

### Confocal microscopy.

Cells were grown on SecureSlip coverslips (Sigma, St. Louis, MO) and fixed with 4% paraformaldehyde, followed by permeabilization with 0.1% Triton X-100 in Hanks balanced salt solution (HBSS) for 10 min. After cells were washed in HBSS, they were incubated with 3% bovine serum albumin (BSA) in HBSS for 1 h to block nonspecific protein adsorption and then incubated with primary antibodies for 2 h at 37°C. After cells were washed three times with HBSS, they were stained for 1 h at 37°C with Alexa Fluor-conjugated secondary antibodies. After samples were washed three times with HBSS, the mounting medium for fluorescence with 4′,6′-diamidino-2-phenylindole (DAPI) was used for staining the nucleus. After being mounted, cells were photographed with a fluorescence confocal microscope (LSM510 META; Zeiss, Germany).

### Microarray data accession number.

The raw and processed microarray data are available in the Gene Expression Omnibus (GEO) database under GEO accession number GSE71665.

## RESULTS

### FAF1 is involved in interferon signaling.

**FIG 1:**
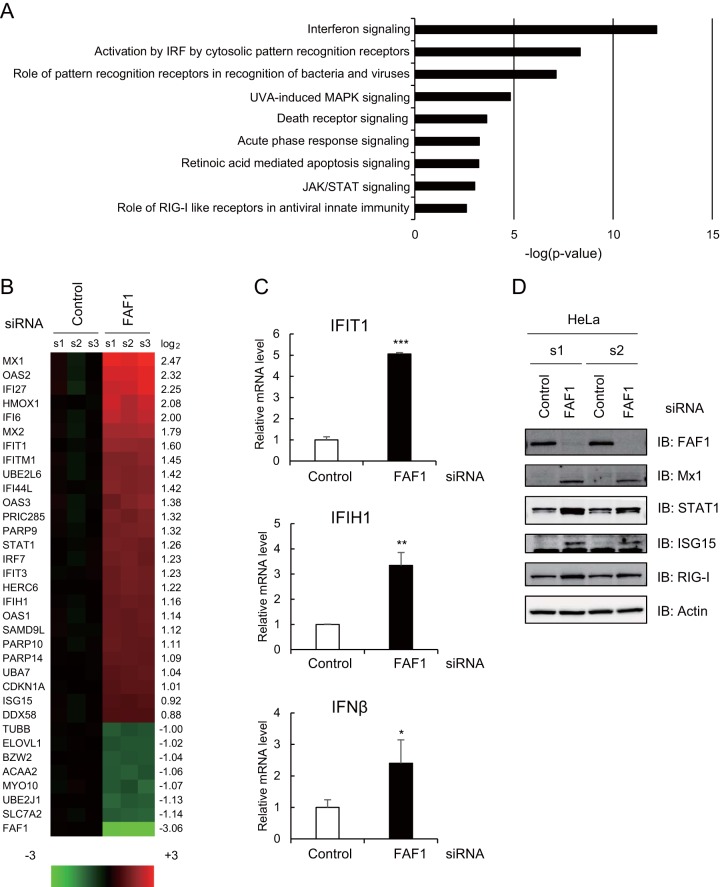
Effects of knocking down FAF1 on the immune signaling pathway. HeLa cells were transfected with a control or FAF1 siRNA 1. After 48 h, cells were harvested and examined. (A and B) Differentially expressed genes were analyzed using a microarray. Genes (fold change of ≥1.5) were analyzed using Ingenuity Pathway Analysis (IPA), and predicted signaling pathways are listed with their *P* values (A). Heat maps (B) show microarray analysis results. Genes up- or downregulated more than 2-fold in FAF1-knockdown cells and some representative genes are listed (left side of the heat map) with their log_2_ ratios (right side of the heat map). Columns s1, s2, and s3 indicate biological triplicates of the experiment. (C) mRNA levels of IFIT1, IFIH1, and IFN-β were measured using RT-qPCR. The values were normalized to GAPDH mRNA values and represent the means ± standard deviations of three experiments. (D) Protein levels of FAF1, Mx1, STAT1, ISG15, and RIG-I were analyzed using Western blot analysis. Actin bands are shown as loading controls. s1 and s2 indicate biological duplicates of the experiment. IB, immunoblot.

To investigate the molecular functions of FAF1, we performed microarray analysis and compared fold changes in mRNA expression levels between control and FAF1-knocked-down HeLa cells. A total 150 genes showed significant changes (fold change of ≥1.5 and *P* value of <0.05) in FAF1-knocked-down HeLa cells compared to control levels. Of these, the expression levels of 66 genes increased, and those of 84 genes decreased in cells in which FAF1 was knocked down. To examine which cellular pathway was mostly affected by silencing FAF1, we conducted Ingenuity Pathway Analysis (IPA) and found that silencing FAF1 significantly raised the expression levels of genes encoding molecules related to IFN signaling and IRF activation (Fig. 1A). Major upregulated genes in FAF1-knocked-down cells are ISGs having antiviral activity, such as Mx1 and Mx2 (Mx1/2), OAS1/2/3, IFIT1/2/3, ISG15, ISG20, and IFI6. Oligoadenylate synthetase (OAS) and Mx genes are the best studied ISGs in terms of antiviral properties. Unlike OAS genes, Mx genes are induced exclusively by IFN-α/β or IFN-δ and are not activated by other cytokines, including interleukin-1 or tumor necrosis factor alpha (TNF-α). Thus, Mx expression has been used as a specific marker for type I IFN induction in clinical settings (32, 33). As shown in Fig. 1B, ISGs increased up to 5.54-fold in FAF1-knocked-down cells. These results suggest that FAF1 suppresses the expression of ISGs by interfering with IFN signaling even in normal cells. To confirm that silencing of FAF1 can induce IFN-β in HeLa cells, we analyzed basal mRNA levels of ISGs and IFN-β using real-time PCR and also measured ISG protein levels using Western analysis. Results shown in Fig. 1 demonstrate that silencing of FAF1 increased the expression levels of the endogenous IFN-β and downstream IFIT1 and IFIH1 (Fig. 1C) and also protein levels of ISGs such as Mx1, STAT1, and ISG15 and RIG-I/DDX58 (Fig. 1D). These results suggest that FAF1 inhibits the expression of IFN-β and downstream genes.

### FAF1 negatively regulates poly(I·C)-induced IFN-β activation.

**FIG 2:**
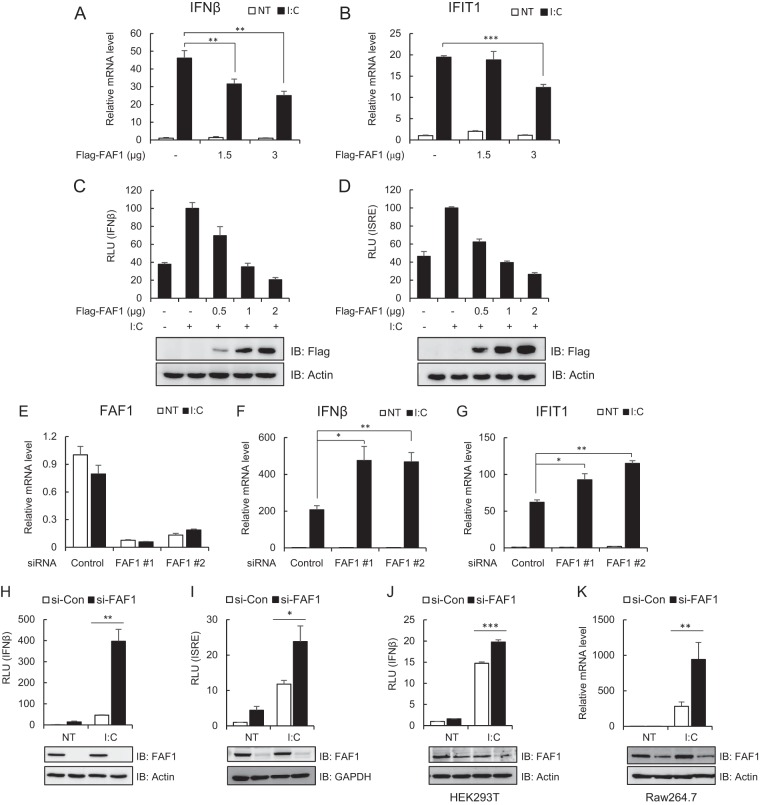
FAF1 inhibits poly(I·C)-induced IFN-β activation. (A and B) HeLa cells were transfected with the indicated amount of Flag-FAF1 plasmid. After 24 h, cells were treated with poly(I·C) (I·C; 10 μg/ml) by transfection for 8 h. mRNA levels of IFN-β and IFIT1 were measured using RT-qPCR. The values were normalized to the value for GAPDH mRNA and represent the means ± standard deviations of three experiments. **, *P* < 0.01; ***, *P* < 0.001 (for differences between Flag and Flag-FAF1 values). NT, not treated. (C and D) HeLa cells were cotransfected with the indicated amount of Flag-FAF1 plasmid and IFN-β-Luc (C) or ISRE-Luc (D) together with a beta-Gal reporter plasmid. After 24 h, cells were treated with poly(I·C) (10 μg/ml) by transfection for 8 h, and relative luciferase activities were measured. The data represent the means ± standard deviations of triplicate experiments. Overexpressed Flag-FAF1 is shown by Western blotting, with actin bands representing loading controls. RLU, relative light units. (E to G) HeLa cells were transfected with two kinds of FAF1 siRNAs, 1 and 2. After 40 h, cells were treated with poly(I·C) (10 μg/ml) by transfection for 8 h. mRNA levels of FAF1, IFN-β, and IFIT1 were measured using RT-qPCR. The values were normalized to the value for GAPDH mRNA. (H and I) HeLa cells were transfected with FAF1 siRNA 2, and 72 h later, cells were transfected with IFN-β-Luc (H) or ISRE-Luc (I) together with a beta-Gal reporter plasmid. Cells were treated with poly(I·C) (10 μg/ml) by transfection for 9 h, and relative luciferase activities were measured. Knockdown of endogenous FAF1 was shown by Western blotting, with actin bands and GAPDH bands representing loading controls. si-CON, control siRNA; si-FAF1, siRNA targeting FAF1. (J) HEK293T cells were transfected with FAF1 siRNA 2 for 72 h and transfected with IFN-β-Luc together with a beta-Gal reporter plasmid. Then cells were treated with poly(I·C) (10 μg/ml) by transfection for 12 h, and relative luciferase activities were measured. (K) Raw264.7 cells were transfected with FAF1 siRNA 2 for 36 h and treated with poly(I·C) (10 μg/ml) for 12 h. The mRNA level of IFN-β was measured using RT-qPCR. The values were normalized to the value for GAPDH. Knockdown of endogenous FAF1 is shown by Western blotting, with actin bands representing loading controls. All of the data represent the means ± standard deviations of three experiments. *, *P* < 0.05; **, *P* < 0.01; ***, *P* < 0.001 (for differences between control siRNA FAF1 siRNA values).

IFN-β is rapidly produced in response to viral infection to induce a cellular antiviral state (5). To investigate whether FAF1 is involved in virus-triggered IFN-β induction, we measured the mRNA level and the IFN-β promoter activity after poly(I·C) transfection in HeLa cells expressing various amounts of FAF1. Transfection with poly(I·C), a synthetic analogue mimicking double-stranded RNA as a stimulant, induces an antiviral response by binding to TLR3 or RLRs. As shown in Fig. 2, overexpression of FAF1 inhibited poly(I·C)-induced transcription of IFN-β and IFIT1 (Fig. 2A and B) and significantly blocked poly(I·C)-induced activation of IFN-β and the interferon-stimulated response element (ISRE) promoter, which is required for expression of the IFN-induced gene, in a dose-dependent manner (Fig. 2C and D). The results demonstrate that FAF1 is a negative regulator of poly(I·C)-triggered IFN-β induction.

Next, we examined poly(I·C)-induced IFN-β using real-time PCR in HeLa cells silencing endogenous FAF1. Two kinds of siRNA constructs were used to block the expression of FAF1 efficiently (Fig. 2E). When the endogenous FAF1 was silenced, transcription levels of IFN-β and IFIT1 increased in response to poly(I·C) stimulation (Fig. 2F and G). Consistent with the above findings, knocking down FAF1 also discernibly increased poly(I·C)-induced activation of IFN-β and the ISRE promoter in HeLa cells (Fig. 2H and I). In order to determine whether this inhibitory effect of FAF1 is cell type specific, we examined FAF1's inhibitory effect in HEK293T (human embryonic kidney cells) and Raw264.7 (mouse macrophage cells) cell lines. Silencing FAF1 in both HEK293T and Raw264.7 cells increased poly(I·C)-induced IFN-β promoter activity and IFN-β transcription as well as in HeLa cells (Fig. 2J and K). These results confirm that FAF1's inhibitory effect on poly(I·C)-induced IFN-β signaling are common in many cell lines tested and not cell type specific.

### Ubiquitin-related domains of FAF1 are not critical for inhibiting IFN-β activation.

**FIG 3:**
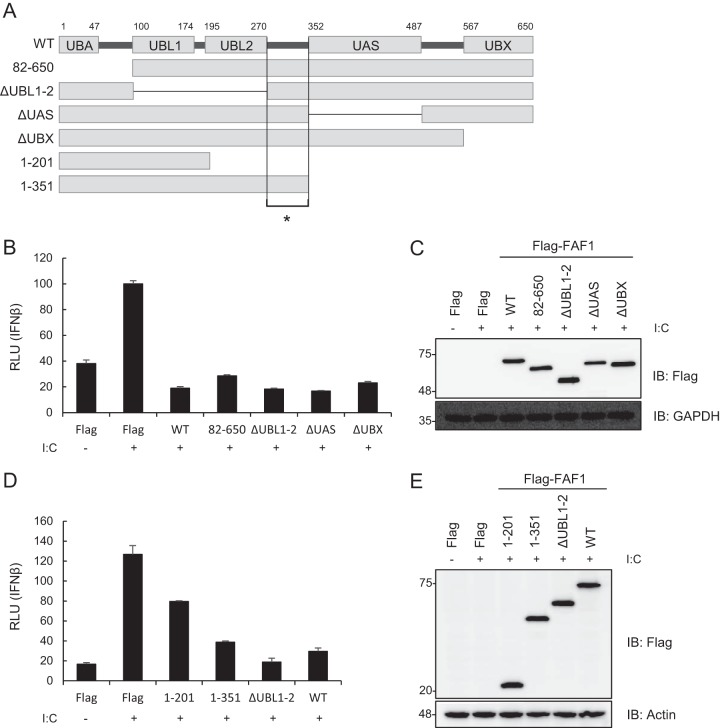
Ubiquitin-related domains of FAF1 are not critical for inhibiting poly(I·C)-induced IFN-β activation. (A) Diagram of various domain deletion mutants of Flag-FAF1. UBA, ubiquitin binding domain; UBL1 and UBL2, ubiquitin-like domains 1 and 2; UAS, upstream activation sequence of unknown function; UBX, ubiquitin regulatory domain. The asterisk indicates the linker region (aa 271 to 351). (B and D) HeLa cells were cotransfected with deletion mutants of Flag-FAF1 plasmid and IFN-β-Luc together with a beta-Gal reporter plasmid. After 24 h, cells were treated with poly(I·C) (10 μg/ml) by transfection for 6 h, and relative luciferase activities were measured. The data represent the means ± standard deviations of triplicate experiments. (C and E) Overexpression of Flag-FAF1 deletion mutants was shown by Western blotting, with GAPDH and actin bands representing loading controls.

In order to identify which of the multiple domains of FAF1 are involved in the inhibition of poly(I·C)-induced IFN-β activation, we measured IFN-β luciferase activity in HeLa cells transfected with wild-type FAF1 and various domain deletion mutants: a deletion of the UBA domain (residues 82 to 650), interacting with polyubiquitinated substrates; a deletion of the UBL1 and UBL2 (ΔUBL1-2) domains interacting with Hsp70; a deletion of the UAS (ΔUAS) domain, whose function is as yet unknown; and a deletion of the UBX (ΔUBX) domain interacting with VCP-Npl4-Ufd1 complex (Fig. 3A). Among the mutants tested, the mutant with a deleted N-terminal UBA domain (aa 82 to 650) and the ΔUBL1-2, ΔUAS, and ΔUBX mutants showed the same inhibitory effects as did wild-type FAF1 (Fig. 3B and C). Since all of these mutants commonly contain two overlapping regions (aa 271 to 351 or aa 488 to 566), we examined which region is involved in the inhibition of IFN-β promoter activity by using truncation mutants consisting of aa 1 to 201, aa 1 to 351, and ΔUBL1-2 and the WT. We found in reporter assays that the expression of aa 1 to 351 and ΔUBL1-2 fully blocked poly(I·C)-induced activation of the IFN-β promoter, while the expression of aa 1 to 201 showed only reduced activation compared to that of the WT (Fig. 3D and E). These results show that the linker region (aa 271 to 351) between UBL2 and the UAS is necessary but not sufficient to inhibit IFN-β promoter activity and that ubiquitin-related domains of FAF1 are not critical for inhibiting IFN-β activation. This finding is unexpected because it is well known that many FAF1 functions are regulated by ubiquitin-related domains.

### Knocking down FAF1 induces antiviral ISG.

**FIG 4:**
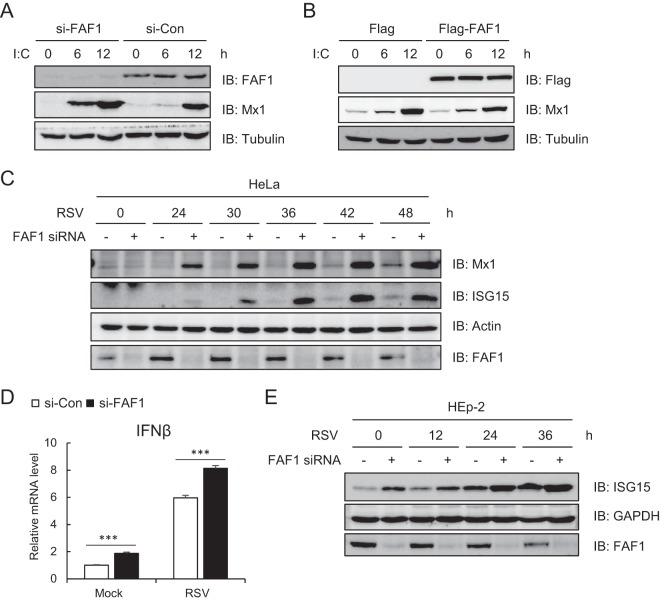
Knocking down FAF1 increased expression of ISGs. (A and B) The level of induced Mx1 was detected by Western blotting, with tubulin bands representing loading controls, in HeLa cells. (A) HeLa cells transfected with a control siRNA or FAF1 siRNA 2 for 40 h were treated with poly(I·C) (10 μg/ml) by transfection for the indicated times. (B) HeLa cells transfected with Flag or Flag-FAF1 for 24 h were treated with poly(I·C) (10 μg/ml) by transfection for the indicated times. (C and E) Levels of ISGs and FAF1 were analyzed by Western blotting, with actin bands representing loading controls, in HeLa cells and HEp-2 cells. Cells were transfected with a control siRNA or FAF1 siRNA 2 for 24 h, and cells were incubated with an RSV inoculum (multiplicity of infection, 1) or uninfected. After 2 h, the medium was changed, and cells were harvested at the indicated time points after infection. (D) Levels of IFN-β mRNA were measured by RT-qPCR in HEp-2 cells silencing FAF1 after RSV infection and then 24 h of incubation. The values were normalized to the value for GAPDH mRNA and represent the means ± standard deviations of three experiments. ***, *P* < 0.001 (for differences between control siRNA and FAF1 siRNA values).

We confirmed the inhibitory effect of FAF1 on IFN-β signaling and on transcription of antiviral ISGs following poly(I·C) stimulation. In addition, silencing FAF1 promoted Mx1 production stimulated by poly(I·C), while overexpressing FAF1 suppressed Mx1 production (Fig. 4A and B). Mx1, a dynamin-like GTPase that broadly inhibits viral replication by trapping viral nucleocapsids, is one of the most highly induced ISGs (34). We next employed respiratory syncytial virus (RSV), a negative-sense, single-stranded RNA virus of the Paramyxoviridae family that causes acute lower respiratory tract infection in young children (35). Like poly(I·C) transfection, silencing FAF1 increased the expression of Mx1 and ISG15 in response to RSV infection in HeLa cells (Fig. 4C). We also investigated whether FAF1 inhibited IFN-β signaling in RSV-infected HEp-2 cells. As shown in Fig. 4D, knocking down FAF1 elevated the transcription of IFN-β and the expression level of ISG15 after RSV infection in HEp-2 cells (Fig. 4E). Collectively, these results suggest that FAF1 inhibits the cellular antiviral response by negatively regulating IFN-β production. We then compared the effect of FAF1 on RSV and vesicular stomatitis virus (VSV) replication by performing plaque formation assays. Silencing FAF1 slightly reduced replication of the viruses, but this inhibitory effect was small, although significant, in VSV compared to the induction of antiviral ISGs (see Fig. S1 in the supplemental material). Further studies are required to prove the influence of FAF1 on viral replication.

### Targets of the FAF1 inhibitory effect during IRF3 activation.

**FIG 5:**
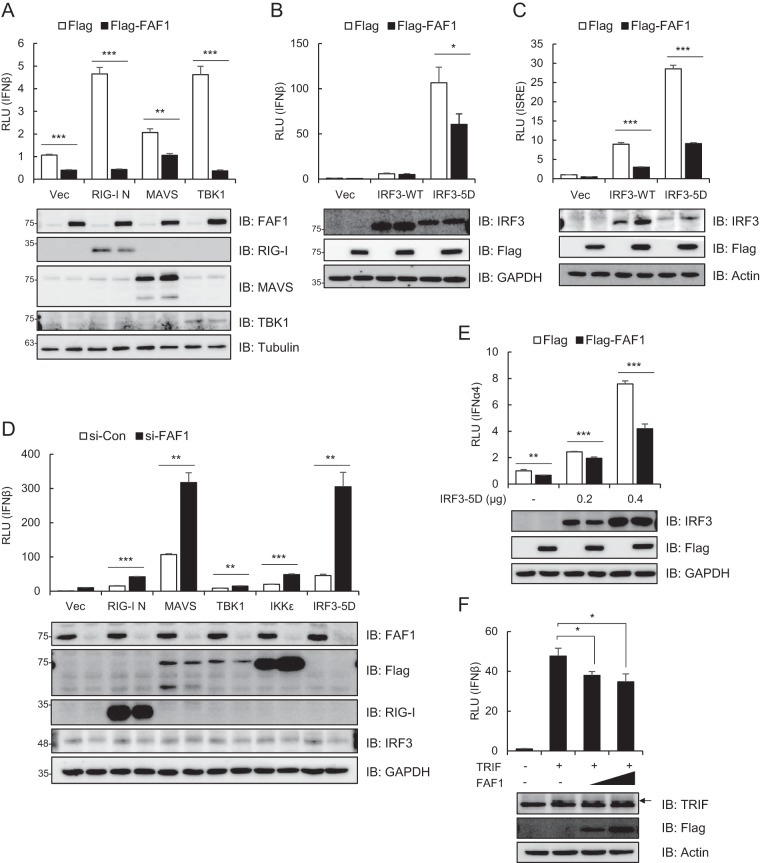
FAF1 operates at or downstream of IRF3. (A) HeLa cells were transfected with IFN-β-Luc, a beta-Gal reporter plasmid, Flag-FAF1, or a control plasmid and Flag-RIG-I N, Flag-MAVS, Flag-TBK1, or a control plasmid (Vec). After 24 h, cells were collected, and relative luciferase activities were measured. The data represent the means ± standard deviations of three experiments. Overexpressed proteins are shown by Western blotting, with tubulin bands representing loading controls. (B and C) HeLa cells were transfected with IFN-β-Luc (B) ISRE-Luc (C), beta-Gal, Flag-FAF1, or a control plasmid and IRF3-WT-GFP, IRF3-5D-GFP, or a control plasmid. At 24 h posttransfection, relative luciferase activities were determined as described for panel A. *, *P* < 0.05; **, *P* < 0.01; ***, *P* < 0.001 (for differences between Flag and Flag-FAF1 values in panels A to C). (D) HeLa cells were transfected with a control siRNA or FAF1 siRNA 2. At 48 h posttransfection, cells were cotransfected with Flag-RIG-I N, Flag-MAVS, Flag-TBK1, Flag-IKKε, IRF3-5D, or a control plasmid and IFN-β-Luc together with a beta-Gal reporter plasmid. At 24 h posttransfection, relative luciferase activities were determined as described for panel A. **, *P* < 0.01; ***, *P* < 0.001 (for differences between values for the control siRNA and FAF1 siRNA). (E) HeLa cells were transfected with IFN-α4-Luc, beta-Gal, Flag-FAF1, or a control plasmid and the indicated amount of IRF3-5D-GFP. At 24 h posttransfection, relative luciferase activities were determined as described for panel A. (F) HeLa cells were transfected with IFN-β-Luc, beta-Gal, Flag-FAF1, or a control plasmid and TRIF. At 24 h posttransfection, relative luciferase activities were determined as described for panel A. *, *P* < 0.05; **, *P* < 0.01; ***, *P* < 0.001 (for differences between values for Flag and Flag-FAF1 in panels E and F).

Recognition of viruses by cytosolic sensors such as RIG-I and MDA5 leads to activation of downstream signaling molecules like MAVS, TBK1, IKKε, and IRF3 (8). To determine the target of the inhibitory effect of FAF1 in the IRF3 activation signaling cascade, we conducted an IFN-β luciferase reporter assay in cells overexpressing each signaling molecule, RIG-I N, MAVS, TBK1, IKKε, or IRF3-5D, together with the IFN-β promoter in the presence and absence of FAF1. RIG-I N is a constitutively active form of RIG-I and is capable of activating IRF3 (36). As shown in Fig. 5A, FAF1 suppressed the activation of IFN-β promoter mediated by overexpression of RIG-I N, MAVS, and TBK1. Furthermore, FAF1 also inhibited IRF3-WT- or IRF3-5D (a constitutively active mutant of IRF3)-induced activation of IFN-β and the ISRE promoter (Fig. 5B and C) even at a higher protein expression level of IRF3-WT or IRF3-5D. These results were confirmed by silencing FAF1, potentiating IFN-β promoter activity mediated by overexpression of all signaling molecules, i.e., RIG-I N, MAVS, TBK1, IKKε, and IRF3-5D (Fig. 5D). The results indicated that FAF1 functions as a negative regulator of IFN-β signaling after phosphorylation of IRF3. Since active IRF3 can bind to the IFN-α4 promoter as well as to IFN-β (37), we additionally used an IFN-α4-Luc plasmid and showed that FAF1 inhibited IRF3-5D-mediated activation of the IFN-α4 promoter (Fig. 5E). Considering that IRF3 could be activated by the TLR3 or TLR4 signaling pathway, we investigated whether FAF1 suppresses the activation of the IFN-β promoter from TLR, employing an IRF3 stimulator, TRIF, an adaptor protein of TLR3 and TLR4. FAF1 also inhibited TRIF-mediated activation of the IFN-β promoter in a dose-dependent manner (Fig. 5F). These results confirm that FAF1 negatively regulates activation of IFN-β at or downstream of IRF3.

### FAF1 does not affect the phosphorylation and dimerization of IRF3.

**FIG 6:**
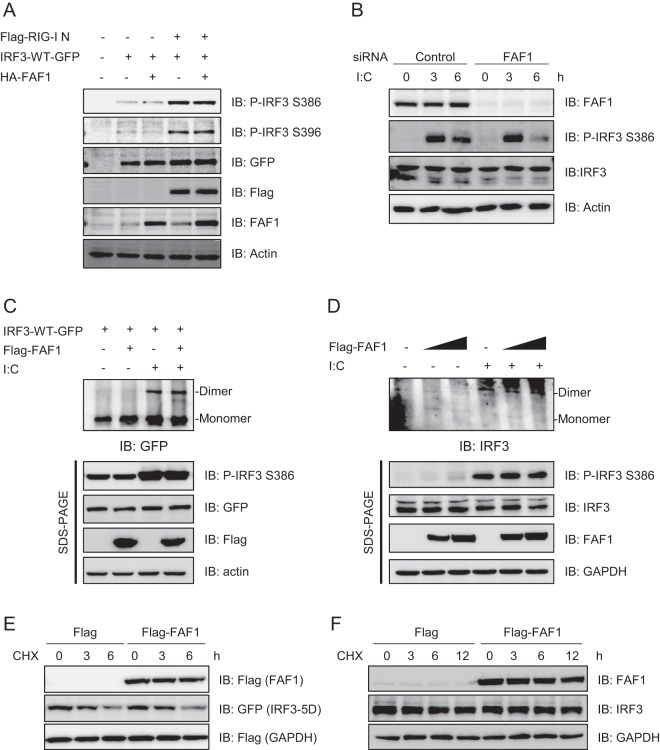
FAF1 does not affect the phosphorylation and dimerization of IRF3. (A) HeLa cells were transfected with Flag-RIG-I N, IRF3-WT-GFP, HA-FAF1, or a control plasmid as indicated. At 24 h posttransfection, cell extracts were analyzed by Western blotting. Actin bands represent loading controls. (B) HeLa cells were transfected with a control siRNA or FAF1 siRNA 2 for 72 h and treated with poly(I·C) (10 μg/ml) by transfection. Cells were then harvested at the indicated time points, and extracts were analyzed by Western blotting, with actin bands representing loading controls. (C) HEK293T cells were cotransfected with IRF3-WT-GFP, Flag-FAF1, or a control plasmid and then stimulated with poly(I·C) (10 μg/ml) for 12 h. Cell extracts were separated by native gel or SDS-PAGE, and IRF3 dimers were detected by Western blotting, with actin bands representing loading controls. (D) HEK293T cells were transfected with Flag-FAF1 or a control plasmid and stimulated with poly(I·C) (10 μg/ml) for 12 h. Endogenous IRF3 was analyzed as described for panel C. (E) HeLa cells were transfected with Flag-FAF1, Flag-GAPDH, IRF3-5D-GFP, or a control plasmid. After 24 h, cells were treated with cycloheximide (50 μg/ml) for the indicated times. Cell lysates were separated by SDS-PAGE and analyzed by Western blotting. (F) HeLa cells were transfected with Flag or Flag-FAF1. After 24 h, cells were treated with cycloheximide (50 μg/ml) for the indicated times. The level of endogenous IRF3 was analyzed by Western blotting, with GAPDH bands representing loading controls.

Upon viral infection, IRF3 is phosphorylated and activated by active TBK1 or IKKε (9). Phosphorylated IRF3 subsequently forms dimers and translocates to the nucleus, where it interacts with transcription coactivators and promotes transcription of IFN-β (10, 11). We investigated whether FAF1 affects phosphorylation and dimerization of IRF3. Using anti-phospho-IRF3 antibody, we detected phosphorylated forms of IRF3 in HeLa cells overexpressing Flag-RIG-I N, IRF3-GFP, and HA-FAF1 or empty vector. As shown in Fig. 6A, RIG-I N overexpression increased the phosphorylation at Ser386 and Ser396 of IRF3; however, FAF1 overexpression did not affect the phosphorylation of IRF3. Consistently, poly(I·C) transfection increased the phosphorylated IRF3, but silencing of FAF1 did not affect the level of phospho-IRF3 (Fig. 6B). These results accorded with the observation that FAF1 repressed the activation of IFN-β promoter induced by the constitutively active mutant IRF3-5D, and thus FAF1 does not affect the phosphorylation of IRF3 in response to stimulation.

Since phosphorylation at Ser386 of IRF3 is required for IRF3 dimerization for nuclear translocation (11), we investigated whether FAF1 inhibits IRF3 dimerization by employing native PAGE. HEK293T cells overexpressing IRF3-WT-GFP with or without Flag-FAF1 were stimulated with poly(I·C), cell lysates were separated by SDS-PAGE or native gel electrophoresis, and dimerization was evaluated by Western blotting as described previously (38). The IRF3 dimers increased in response to poly(I·C) transfection on a native gel; however, no discernible change in dimerization of IRF3 was detected in cells overexpressing FAF1. Phospho-IRF3 at Ser386 was also detected in cells stimulated by poly(I·C) regardless of FAF1 expression on SDS-PAGE gels (Fig. 6C). We also investigated the effect of FAF1 overexpression on the dimerization of endogenous IRF3 in response to poly(I·C) stimulation and found that FAF1 did not affect the dimerization of endogenous IRF3 (Fig. 6D). Taken together, these results demonstrate that FAF1 inhibits neither phosphorylation nor dimerization of IRF3 but may inhibit some downstream event after activation of IRF3.

Since we previously found that FAF1 is a ubiquitin receptor that facilitates the degradation of Hsp70 and ERAD substrates by the ubiquitin proteasome system (26, 29, 30), we investigated whether FAF1 affects the degradation of IRF3. We monitored the half-lives of endogenous IRF3 and the active mutant IRF3-5D after the cells were treated with cycloheximide (CHX), an inhibitor of protein synthesis. Overexpression of FAF1 did not affect the degradation of IRF3 (Fig. 6E and F).

### FAF1 interferes the interaction between IRF3 and IPO5.

**FIG 7:**
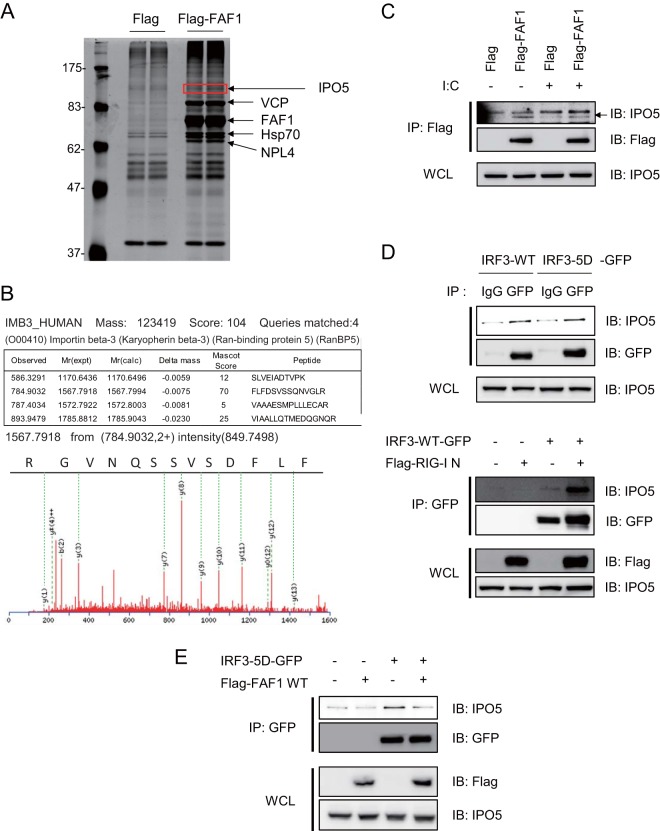
FAF1 overexpression abolishes IRF3-IPO5 interaction. (A and B) Silver gel and MS/MS spectrum of IPO5 which was detected in FAF1 immune complex and identified employing nano-UPLC-ESI-q-TOF tandem MS. (C) HeLa cells transfected with Flag or Flag-FAF1 were treated with poly(I·C) (10 μg/ml) for 6 h, and immunoprecipitation of FAF1 was performed using anti-Flag antibody. Immune complexes and whole-cell lysates (WCL) were separated by SDS-PAGE and analyzed by Western blotting. (D) HEK293T cells were transfected with IRF3-WT-GFP or IRF3-5D-GFP. After 30 h, cell lysates were immunoprecipitated with anti-GFP antibody or anti-IgG antibody, and immune complexes were analyzed by Western blotting (upper panel). HEK293T cells were transfected with IRF3-WT-GFP, Flag-RIG-I N, or a control plasmid as indicated. After 30 h, cell lysates were immunoprecipitated with anti-GFP antibody, and immune complexes were analyzed by Western blotting (lower panel). (E) HEK293T cells were cotransfected with IRF3-5D-GFP, Flag-FAF1, or a control plasmid as indicated. After 30 h, cells lysates were immunoprecipitated with anti-GFP antibody, and immune complexes were analyzed by Western blotting.

In the inhibition of IRF3-5D-mediated activation of IFN-β promoter by FAF1, no physical interaction between FAF1 and IRF3 in control or stimulated cells was detected (data not shown). One explanation for this is that FAF1 may inhibit IRF3-driven IFN-β signaling via an adaptor protein. In order to find the adaptor molecule of FAF1 in IRF3 inhibition, we performed immunoprecipitation with anti-Flag antibody in HeLa cells overexpressing Flag-FAF1; immune complexes were separated by SDS-PAGE and detected with silver staining (Fig. 7A). IPO5/importin-β3 was identified as a protein physically associated with FAF1 by peptide sequencing with nano-ultraperformance liquid chromatography with electrospray ionization-quadrupole time of flight (nano-UPLC-ESI-q-TOF) tandem MS (Fig. 7B) and confirmed by Western blotting (Fig. 7C). IPO5/importin-β3 was first identified as a binding protein of a small Ran GTPase. It is a member of the importin-β family which binds directly to cargo proteins or to importin-α and cargo complex to promote the nuclear import of cargo proteins (39). A recent study shows that IPO5 interacts with the transcription factor β-catenin and plays a role in nuclear transport of β-catenin (40). IRF3 is known to be a subset of importin-α receptors, and FAF1, inhibiting IRF3-mediated IFN-β activation, interacts with IPO5 in control and poly(I · C)-stimulated cells (Fig. 7C). Hypothesizing that FAF1 interferes with the translocation of IRF3 to the nucleus by inhibiting the interaction between IRF3 and IPO5, we investigated whether IRF3-5D binds to IPO5 and found such interaction in HEK293T cells overexpressing IRF3-5D-GFP. When the cell lysates were immunoprecipitated with anti-GFP antibody, we found that IRF3-5D indeed interacted with IPO5 (Fig. 7D, upper panel). Furthermore, the interaction between IRF3-WT and IPO5 was increased in response to overexpression of RIG-I N. These results showed that activated IRF3 more strongly interacted with IPO5 (Fig. 7D, lower panel).

We then tested whether FAF1 inhibits IFN-β signaling through competition with IRF3 for IPO5 binding. HEK293T cells cotransfected with IRF3-5D-GFP and Flag-FAF1 were lysed and immunoprecipitated with anti-GFP antibody. As shown in Fig. 7E, IRF3-5D physically associates with IPO5, and the amount of IPO5 interacting with IRF3-5D was reduced by FAF1 overexpression. In Western blot analysis with whole-cell lysates, both IRF3-5D and FAF1 were well expressed, and the levels of IPO5 were not different between the lanes. Although FAF1 and IRF3 did not directly interact with each other, FAF1 can inhibit IFN-β activation by disturbing the interaction between active IRF3 and IPO5.

### FAF1 suppresses nuclear translocation of IRF3.

**FIG 8:**
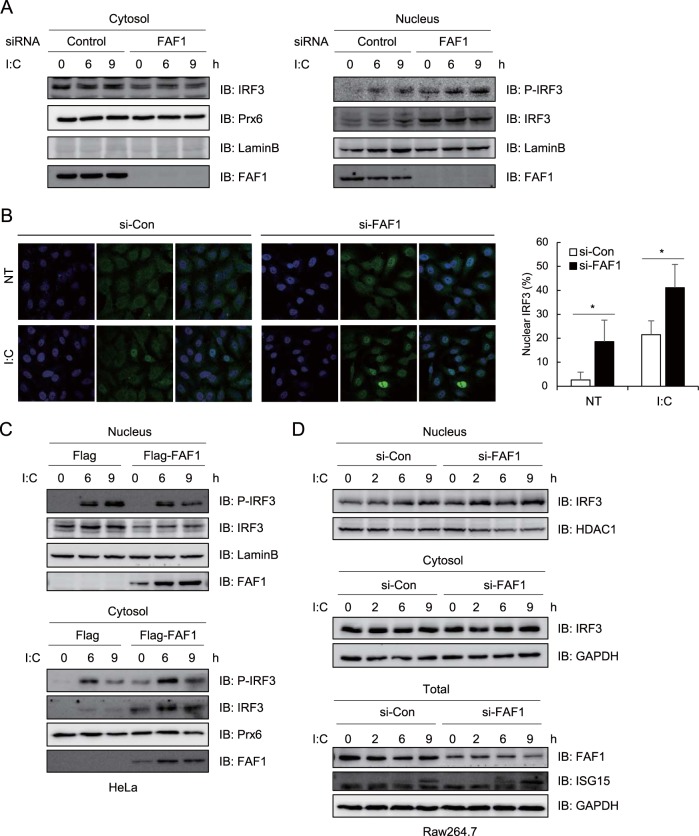
Knocking down FAF1 promotes nuclear translocation of IRF3. HeLa cells were transfected with a control siRNA or FAF1 siRNA 2 for 72 h. (A) Cells were treated with poly(I·C) (10 μg/ml) by transfection for the indicated times, and then lysates were divided into cytosolic and nuclear fractions as described in Materials and Methods. Nuclear translocation of IRF3 was assayed using Western blot analysis. Prx6 and lamin B were used as cytosolic and nuclear markers, respectively. (B) Localization of IRF3 in HeLa cells was evaluated by fluorescence confocal microscopy. Control and FAF1-knocked-down HeLa cells were treated with a control and poly(I·C) (10 μg/ml) for 3 h. Then cells were fixed, permeabilized, and stained with anti-IRF3 antibody (green). Nuclei were detected with DAPI staining (blue). The graph indicates the percentages of cells showing nuclear immunoreactivity for IRF3. Data were calculated after counting the number of cells with nuclear IRF3 from more than 5 fields from the coverslips using ImageJ software (right panel). *, *P* < 0.05 (for the difference between the values for the control siRNA and FAF1 siRNA). (C) HeLa cells were transfected with FAF1 siRNA 2 for 40 h and transfected with Flag or Flag-FAF1. At 24 h posttransfection, cells were treated with poly(I·C) (10 μg/ml) by transfection for the indicated times; cell lysates were divided by fractionation and analyzed as described for panel A. (D) Raw264.7 cells were transfected with a control siRNA or FAF1 siRNA 2 for 40 h, and cells were treated with poly(I·C) (10 μg/ml) for the indicated times. Total samples were obtained before centrifugation, and then cell lysates were divided by fractionation. GAPDH and HDAC1 were used as cytosolic and nuclear markers, respectively.

FAF1 blocks IRF3-5D-induced IFN-β promoter activation without affecting the phosphorylation or dimerization of IRF3. Since FAF1 inhibited IRF3-IPO5 interaction, we investigated whether FAF1 is involved in nuclear translocation of IRF3. We stimulated control and FAF1-knocked-down HeLa cells with poly(I·C), isolated the nuclear fraction, and assessed the amount of IRF3 that translocated into the nucleus in response to poly(I·C) using anti-phospho-IRF3 antibody. We found that nuclear IRF3 significantly increased (Fig. 8A, right panel), while cytosolic IRF3 slightly decreased in cells knocking down FAF1 compared to levels in control cells (Fig. 8A, left panel). This translocation to the nucleus was confirmed with fluorescence microscopy of IRF3 in the FAF1-knocked-down cells treated with poly(I·C). Image analysis showed translocation of IRF3 into the nucleus, and counting cells showing nuclear IRF3 confirmed that silencing FAF1 promoted translocation of IRF3 to the nucleus in both control and poly(I·C)-transfected cells (Fig. 8B). Next, in order to investigate whether overexpressed FAF1 could inhibit nuclear translocation of IRF3, we assessed IRF3 in the nucleus of HeLa cells. To maximize the effect, HeLa cells knocking down endogenous FAF1 with an siRNA were transfected with Flag or Flag-FAF1 and stimulated with poly(I·C). Nuclear and cytosolic fractions were obtained by fractionation. IRF3 and phospho-IRF3 in the nuclear fraction were decreased, and levels in the cytosolic fraction increased in cells overexpressing FAF1 compared to levels in control cells (Fig. 8C). The results were also confirmed in mouse Raw 264.7 cells. As shown in Fig. 8D, FAF1 was not completely knocked downed in the Raw264.7 cells, so the reduction of cytosolic IRF3 was not dramatic. But nuclear IRF3 was accumulated more, and the expression of ISG15 was increased in cells knocking down FAF1 compared to levels in control cells.

**FIG 9:**
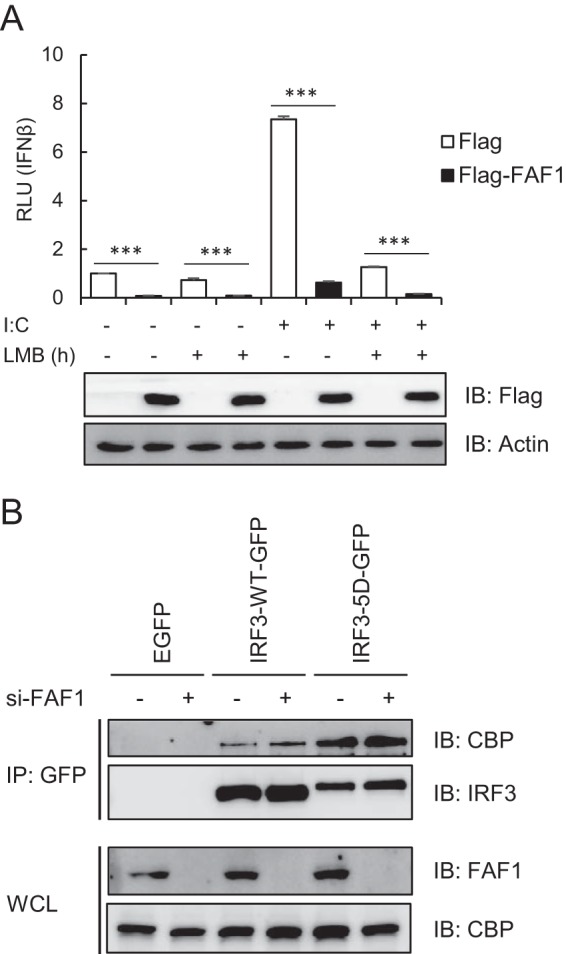
FAF1 does not affect nuclear export of IRF3 and recruitment of CBP to IRF3. (A) HeLa cells were cotransfected with IFN-β-Luc, beta-Gal, Flag-FAF1, or a control plasmid. After 24 h, cells were treated with poly(I·C) (10 μg/ml) and incubated in the presence or absence of LMB (20 nM). After 6 h of incubation, cells were harvested, and relative luciferase activities were measured. The data represent the means ± standard deviations of three experiments. ***, *P* < 0.001 (for differences between values for Flag and Flag-FAF1). (B) HeLa cells were transfected with a control siRNA or FAF1 siRNA 2 for 48 h and then transfected with enhanced GFP, IRF3-WT-GFP, or IRF3-5D-GFP. At 24 h posttransfection, cell lysates were immunoprecipitated with anti-GFP antibody, and immune complexes were analyzed by Western blotting.

Nuclear accumulation of IRF3 could occur by impairment of nuclear export. IRF3 has an active nuclear export signal (NES), and its nuclear export depends on the activity of CRM1 (23). To test whether nuclear export of IRF3 was affected by FAF1, we examined IFN-β promoter activity after overexpressing FAF1 in the absence or presence of a specific CRM1 inhibitor, leptomycin B (LMB). As shown in Fig. 9A, FAF1 inhibited poly(I·C)-induced IFN-β promoter activity regardless of LMB treatment. These findings indicate that FAF1 regulates IRF3 translocation to the nucleus by modulating the nuclear import step, not the export step.

In order to investigate whether FAF1 affects the interaction of active IRF3 with the coactivators CBP/p300, we examined the interaction of CBP with IRF3-WT and IRF3-5D in presence and absence of FAF1. Interaction of active IRF3-5D with CBP significantly increased, but FAF1 did not affect this interaction (Fig. 9B). This indicates that FAF1 does not affect the interaction between nuclear active IRF3 and CBP; rather FAF1 affects the translocation of IRF3 from cytosol to nucleus.

## DISCUSSION

FAF1, a member of the UBXN family containing the UBA-UBX domain, plays multiple biological functions, including protein degradation of Hsp70 and ERAD substrate (26, 28, 30). However, the functions of FAF1 remain to be understood. We screened target genes of FAF1 by employing microarray analysis. Our microarray studies of FAF1-knocked-down HeLa cells showed that FAF1 changed the expression levels of 150 genes, including genes related to apoptosis and proteolysis. The genes showing the most significant changes by FAF1 depletion were ISGs which encode proteins repressing viral replication or enhancing type I IFN production.

This study shows that overexpression of FAF1 inhibited poly(I·C)-induced activation of IFN-β and ISRE promoter as well as poly(I·C)-induced transcription of IFN-β and IFIT1 and ISG expression. Silencing FAF1 increased IFN-β production in response to poly(I·C) stimulation in all cell lines tested. Simultaneously, silencing of FAF1 promoted RSV-induced IFN-β production and substantial induction of antiviral ISGs such as Mx1 and ISG15. Reporter assays demonstrated that FAF1 functions at the downstream step of IRF3 phosphorylation. FAF1 did not alter phosphorylation and dimerization of IRF3 (Fig. 6); rather, silencing FAF1 augmented its nuclear translocation.

To understand how FAF1 regulates the innate immune system by modulating IFN-β activation, we performed IFN-β reporter assays using molecules in both the RLR and TLR signaling axes. These studies revealed that FAF1 suppressed activation of the IFN-β promoter, downstream of IRF3 phosphorylation. Silencing FAF1 potentiated IFN-β activation mediated by overexpression of RIG-I N, MAVS-, TBK1-, IKKε, and IRF3-5D. Neither overexpression nor knockdown of FAF1 affected the phosphorylation and dimerization of IRF3. We also examined whether FAF1 facilitates the degradation of IRF3 by the ubiquitin proteasome system because FAF1 is known as a ubiquitin receptor protein. However, FAF1 overexpression did not increase the degradation of endogenous IRF3 or its active mutant. In addition, a UBA domain deletion mutant which could not interact with polyubiquitinated substrates also inhibited poly(I·C)-induced IFN-β activation as well as wild-type FAF1. Then, we examined whether FAF1 affects the cellular localization of IRF3 and found that silencing FAF1 increased the accumulation of active IRF3 in the nucleus induced by poly(I·C) transfection, while overexpression of FAF1 reduced the levels of nuclear IRF3 upon poly(I·C) transfection. These results indicate that FAF1 inhibits IRF3 translocation to the nucleus, not its phosphorylation or degradation.

**FIG 10:**
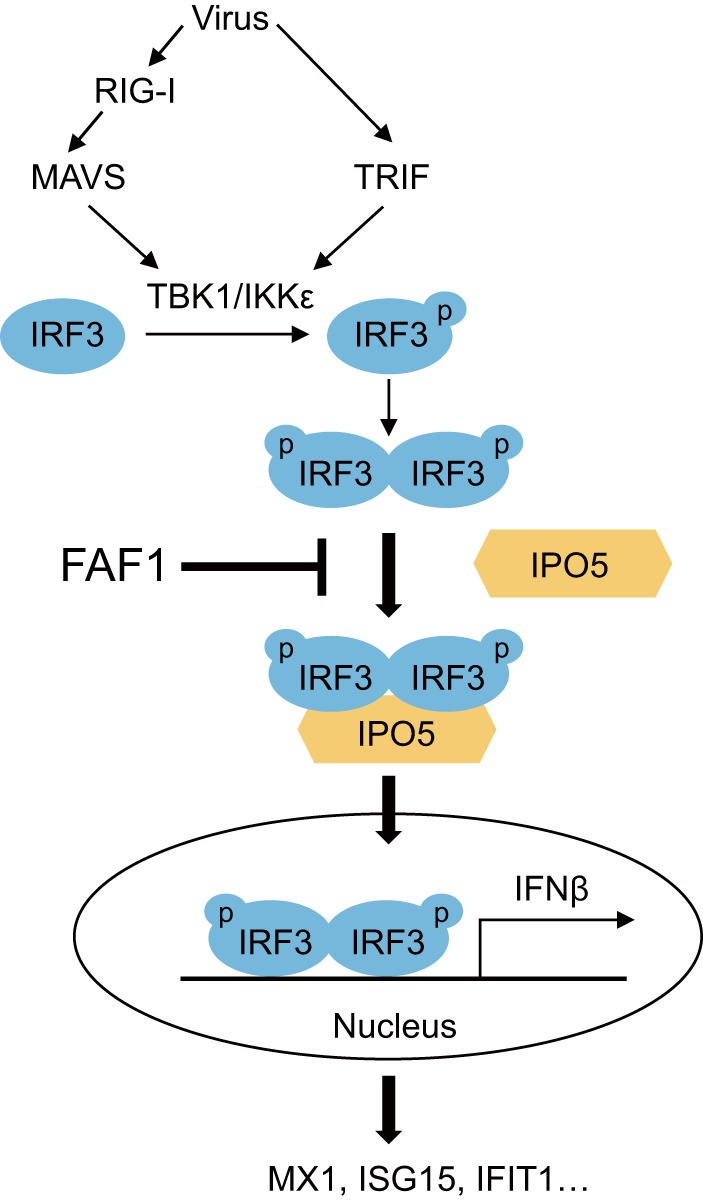
Schematic diagram of FAF1 action on the innate-immunity signaling pathway. Virus infection recruits the kinases TBK1 and IKKε to adaptor proteins MAVS and TRIF. These kinases phosphorylate (p) IRF3, and phosphorylated IRF3 forms dimers which translocate into the nucleus via interaction with IPO5 to produce IFN-β. However, FAF1 inhibits IFN-β activation and ISG induction by interfering with the IRF3-IPO5 interaction and represses nuclear translocation of IRF3.

How does FAF1 regulate the nuclear translocation of IRF3? Previous studies have shown that the IRF3 NLS and nuclear localization of IRF3 are important for IRF3 transcriptional activity (21, 22). IRF3 is known to be phosphorylated by upstream kinases, forming dimers which are then transported into the nucleus (8, 9). In order to identify how FAF1 inhibits IRF3 nuclear translocation, we examined the interacting proteins of FAF1 and found for the first time that FAF1 constitutively interacted with the nuclear import receptor IPO5/importin-β3 and that active IRF3 also associated with IPO5/importin-β3 (Fig. 10).

There are many nuclear import receptors, including 7 importin-α genes and 20 importin-β genes in human (41). Proteins greater than 40 kDa are known to pass through the nuclear pore complex using receptors and carrier proteins. Until now, the correlations between cargo proteins and importin receptor have not been well identified, and many cargoes use more than one import factor for translocation. IPO5/importin-β3, interacting with FAF1 and IRF3 (Fig. 7C and D), is a member of the importin-β family and binds to cargo directly without importin-α adaptor and mediates nuclear import of ribosomal proteins, histones, and viral proteins (39, 42). Several viruses are known to escape the immune response by inhibiting nuclear import of transcription factors. The Ebola virus VP24 protein inhibits interaction of STAT1 with importin-α1 (43). Hepatitis B virus polymerase interrupts STAT1/2 binding to importin-α5 (44). Until the present study, IRF3 was known to interact with importin-α3 and importin-α4. This study shows that IRF3 associates with IPO5/importin-β3, and this interaction is increased by overexpression of RIG-I N, which leads to activation of IRF3 (Fig. 7D, lower panel). Since no direct interaction between IRF3 and FAF1 was observed, we propose that interaction between FAF1 and IPO5 could affect nuclear import of IRF3. We found that overexpressing FAF1 significantly decreased the interaction between IRF3 and IPO5 (Fig. 5E). This is a novel inhibitory mechanism by which FAF1 regulates IRF3-mediated IFN-β induction. Further studies are needed to understand how FAF1 inhibits innate immunity, including interacting with nuclear pore complex components such as Ran GTPase and other importin receptors.

In summary, this study suggests that FAF1 plays a key role as a negative regulator of the innate immune system in general and of virus-triggered IFN-β production in particular, including inhibiting the translocation of IRF3 into the nucleus and preventing antiviral IFN-β signaling. This is a novel biological function to be added to the list of cellular functions of FAF1. FAF1 can be a valuable target for developing therapeutics of autoimmune and inflammatory diseases caused by IFN-β.

## Supplementary Material

Supplemental material
