# Supplementary material for: Fas-Associated Factor 1 Negatively Regulates the Antiviral Immune Response by Inhibiting Translocation of Interferon Regulatory Factor 3 to the Nucleus
Source: Mol Cell Biol. 2016 Mar 18;36(7):1136–51. doi: 10.1128/MCB.00744-15 (PMC4800795; doi:10.1128/MCB.00744-15)
Supplement: Supplemental material [file supp_36_7_1136__index.html]

Supplemental material 

# Fas-Associated Factor 1 Negatively Regulates the Antiviral Immune Response by Inhibiting Translocation of Interferon Regulatory Factor 3 to the Nucleus

## Supplemental material

- Supplemental file 1 -

  Table S1 (Genes with >1.5-fold change in FAF1 knockdown) and Fig. S1 (Plaque formation assay using VSV and RSV)

  PDF, 7.5M
