# Supplementary material for: Fas-Associated Factor 1 Negatively Regulates the Antiviral Immune Response by Inhibiting Translocation of Interferon Regulatory Factor 3 to the Nucleus
Source: Mol Cell Biol. 2016 Mar 18;36(7):1136–51. doi: 10.1128/MCB.00744-15 (PMC4800795; doi:10.1128/MCB.00744-15)
Supplement: Supplemental material [file MCB.00744-15_zmb999101183so1.pdf]

Supplementary Table 1. Genens with fold change greater than 1.5 in FAF1 knock down compared to control knock down HeLa cells

Gene Name	Gene Symbol	**Fold change (FAF1/ CTL)**	Accession No.
MX dynamin-like GTPase 1	MX1	**5.549**	NM_002462.2
2'-5'-oligoadenylate synthetase 2	OAS2	**4.990**	NM_016817.2
Interferon alpha-inducible protein 27, mitochondrial	IFI27	**4.766**	NM_005532.3
Heme oxygenase 1	HMOX1	**4.236**	NM_002133.1
interferon, alpha-inducible protein 6	IFI6	**3.991**	NM_022872.2
MX dynamin-like GTPase 2	MX2	**3.464**	NM_002463.1
Interferon-induced protein with tetratricopeptide repeats 1	IFIT1	**3.035**	NM_001548.3
Interferon alpha-inducible protein 6	IFI6	**2.918**	NM_022873.2
Interferon-induced transmembrane protein 1	IFITM1	**2.739**	NM_003641.3
Ubiquitin/ISG15-conjugating enzyme E2 L6	UBE2L6	**2.681**	NM_004223.3
Interferon-induced protein 44-like	IFI44L	**2.678**	NM_006820.1
2'-5'-oligoadenylate synthase 3	OAS3	**2.609**	NM_006187.2
Helicase with zinc finger domain 2	PRIC285	**2.500**	NM_033405.2
Poly [ADP-ribose] polymerase 9	PARP9	**2.490**	NM_031458.1
Signal transducer and activator of transcription 1-alpha/beta	STAT1	**2.399**	NM_007315.2
cDNA clone IMAGE:6156595 5-, mRNA sequence		**2.364**	BQ437417
Interferon regulatory factor 7	IRF7	**2.353**	NM_004029.2
Interferon-induced protein with tetratricopeptide repeats 3	IFIT3	**2.347**	NM_001031683.1
Probable E3 ubiquitin-protein ligase HERC6	HERC6	**2.333**	NM_017912.3
Gamma-interferon-inducible protein 16	IFIH1	**2.236**	NM_022168.2
2'-5'-oligoadenylate synthase 1	OAS1	**2.211**	NM_001032409.1
SAM domain-containing protein 9-like	SAMD9L	**2.179**	NM_152703.2
Poly [ADP-ribose] polymerase 10	PARP10	**2.164**	NM_032789.1
Poly [ADP-ribose] polymerase 14	PARP14	**2.122**	NM_017554.1
Signal transducer and activator of transcription 1-alpha/beta	STAT1	**2.087**	NM_007315.2
2'-5'-oligoadenylate synthase 1	OAS1	**2.086**	NM_001032409.1
Ubiquitin-like modifier-activating enzyme 7	UBA7	**2.063**	NM_003335.2
Cyclin-dependent kinase inhibitor 1A	CDKN1A	**2.010**	NM_000389.2
	LOC10012827[illegible]	**1.968**	XM_001725558.1
Interferon regulatory factor 7	IRF7	**1.951**	NM_004029.2
Growth differentiation factor 15	GDF15	**1.945**	NM_004864.1
Bcl2 modifying factor	BMF	**1.934**	NM_033503.3
Interferon regulatory factor 9	IRF9	**1.913**	NM_006084.4
Signal transducer and activator of transcription 1-alpha/beta	STAT1	**1.912**	NM_139266.1
2'-5'-oligoadenylate synthase 1	OAS1	**1.909**	NM_002534.2
Ubiquitin-like protein ISG15	ISG15	**1.893**	NM_005101.1
Probable ATP-dependent RNA helicase DDX58	DDX58	**1.843**	NM_014314.3
Poly [ADP-ribose] polymerase 9	PARP9	**1.833**	NM_031458.1

Carbonic anhydrase 9	CA9	1.823	NM_001216.1
Meiotic recombination protein REC8 homolog	REC8	1.815	NM_005132.2
Phospholipid scramblase 1	PLSCR1	1.790	NM_021105.1
Complement component1	C1S	1.703	NM_001734.2
Sialidase 1	NEU1	1.688	NM_000434.2
Ferritin, heavy polypeptide 1 pseudogene 12	FTHL12	1.669	NR_002205.1
Ferritin, heavy polypeptide 1 pseudogene 3	FTHL3	1.657	NR_002201.1
Signal transducer and activator of transcription 2	STAT2	1.633	NM_005419.2
Caspase1	CASP1	1.627	NM_033294.2
Interferon-induced protein with tetratricopeptide repeats 3	IFIT3	1.619	NM_001549.2
Plasminogen activator inhibitor 1	SERPINE1	1.613	NM_000602.1
Poly [ADP-ribose] polymerase 12	PARP12	1.607	NM_022750.2
ATP-binding cassette subfamily A member 1	ABCA1	1.605	NM_005502.2
UPF0515 protein C19orf66	C19orf66	1.602	NM_018381.2
Complement factor B	CFB	1.600	NM_001710.4
Interferon-induced 35KDa protein	IFI35	1.600	NM_005533.2
	LOC729009	1.588	XR_042330.1
Caspase 1	CASP1	1.585	NM_033294.2
Poly [ADP-ribose] polymerase 10	PARP10	1.570	XM_001127571.1
Ferritin, heavy polypeptide 1 pseudogene 2	FTHL2	1.570	NR_002200.1
Ferritin, heavy polypeptide 1 pseudogene 8	FTHL8	1.557	NR_002203.1
Bcl-2 modifying factor	BMF	1.552	NM_001003943.1
Probable E3 ubiquitin-protein ligase HERC 5	HERC5	1.549	NM_016323.2
Steril alpha motif domain containing 9	SAMD9	1.540	NM_017654.2
Interferon-induced protein with tetratricopeptide repeats 2	IFIT2	1.526	NM_001547.4
Interferon-stimulated gene 20kDa protein	ISG20	1.521	NM_002201.4
TPR and ankyrin repeat-containing protein 1	LBA1	1.516	NM_014831.1
Guanylate binding protein 2	GBP2	1.513	NM_004120.3
Gamma-interferon-inducible protein 16	FTHL11	1.512	NR_002204.1
	LOC642567	1.509	XR_038054.1
Gamma-interferon-inducible protein 16	IFI16	1.506	NM_005531.1
PHD finger protein 11	PHF11	1.503	NM_001040443.1
Ferritin, heavy polypeptide 1 pseudogene 12	FTHL12	1.503	NR_002205.1
Interluekin 1 beta	IL1B	1.502	NM_000576.2
	LOC731049	-1.503	XM_001129232.1
cDNA FLJ31090 fis, clone IMR321000102		-1.504	AK055652
Kruppel-like factor 2	KLF2	-1.505	NM_016270.2
Aquaporin 3	AQP3	-1.505	NM_004925.3
Major facilitator superfamily domain-containing protein 6	MFSD6	-1.509	NM_017694.3
Inhibitor of growth protein 1	ING1	-1.510	NM_198219.1
Methionine sulfoxide reductase B3	MSRB3	-1.511	NM_198080.2
TRIO and Factin binding protein	TRIOBP	-1.512	NM_138632.2
La ribonucleo protein domain family member 7	LARP7	-1.515	NM_015454.1

Nucleophosmin/nucleoplasmin 3	NPM3	-1.524	NM_006993.1
Membrane associated ring finger 4	MARCH4	-1.530	NM_020814.1
Pleckstrin homology domain containing family A member 1	PLEKHA1	-1.533	NM_001001974.1
Pyruvate dehydrogenase alpha1	PDHA1	-1.533	NM_000284.1
Sodium channel voltae gated type 9 alpha subunit	SCN9A	-1.536	NM_002977.2
Tripeptityl peptidase1	TPP1	-1.540	NM_000391.3
SLC2A4 regulator	SLC2A4RG	-1.544	NM_020062.3
Tocopherol transfer protein-like	TTPAL	-1.546	NM_024331.3
LIM homeobox 6	LHX6	-1.550	NM_199160.2
Transmembrane protein 117	TMEM117	-1.551	NM_032256.1
Protein kinase C iota type	PRKCI	-1.551	NM_002740.5
Alpha-mannosidase 2	MAN2A1	-1.552	NM_002372.2
Tumor protein D52	TPD52	-1.552	NM_005079.2
FERM domain containing 3	FRMD3	-1.553	NM_174938.3
Ras-related GTP binding C	RRAGC	-1.554	NM_022157.2
Nudix type motif 1	NUDT1	-1.555	NM_198948.1
Coagulation factor XII	F12	-1.566	NM_000505.3
Translocator protein	TSPO	-1.572	NM_007311.3
Vesicular, overexpressed in cancer, prosurvival protein 1	ECOP	-1.576	NM_030796.2
Insulin-like growh factor binding protein 5	IGFBP5	-1.581	NM_000599.2
Adaptor molecule crk	CRK	-1.589	NM_016823.2
Hairy and enhancer of split 6	HES6	-1.589	NM_018645.3
full length insert cDNA clone EUROIMAGE 966164		-1.597	AJ420516
Rho GTPase activatin protein 19	ARHGAP19	-1.597	NM_032900.4
Trans-Golgi network integral membrane protein 2	TGOLN2	-1.602	NM_006464.2
Basic leucine zipper transcription factor	BATF3	-1.603	NM_018664.1
	LOC375295	-1.604	XM_374020.4
Serine/threonine-protein kinase mTOR	FRAP1	-1.607	NM_004958.2
Solute carrier family 16 member14	SLC16A14	-1.616	NM_152527.3
Glutaminase	GLS	-1.618	NM_014905.2
Transmembrane protein 2	TMEM2	-1.619	NM_013390.1
Serine/arginine-rich splicing factor 7	SFRS7	-1.623	NM_001031684.1
Family with sequence similarity 83 member A	FAM83A	-1.629	NM_032899.4
GTP-binding protein SAR1b	SAR1B	-1.633	NM_001033503.1
Bcl10-interacting CARD protein	C9orf89	-1.637	NM_032310.3
E3 ubiquitin-protein ligase NEDD4-like	NEDD4L	-1.639	NM_015277.3
Sorbitol dehydrogenase	SORD	-1.640	XM_001132175.1
Junctophilin1	JPH1	-1.645	NM_020647.2
cDNA FLJ42306 fis, clone TRACH2001646		-1.646	AK124299
G protein pathway suppressor 1	GPS1	-1.650	NM_004127.4
Small acidic protein	C11orf58	-1.653	NM_001142705.1
Neuroblast differentiation-associated protein AHNAK	AHNAK	-1.655	NM_001620.1
Ras-related protein Rab-6A	RAB6A	-1.685	NM_002869.4

Calcium-activated potassium channel subunit alpha-1	KCNMA1	-1.686	NM_002247.2
MAP7 domain containing	MAP7D2	-1.703	NM_152780.2
Calcium/calmodulin-dependent protein kinase inhibitor 1	CAMK2N1	-1.710	NM_018584.5
Delta-like 3	DLL3	-1.716	NM_016941.2
Growth-regulated alpha protein	CXCL1	-1.717	NM_001511.1
Cyclin Y	CCNY	-1.718	NM_145012.3
Caspase7	CASP7	-1.721	NM_033339.3
MAGUK p55 subfamily member 5	MPP5	-1.729	NM_022474.2
Sorbitol dehydrogenase	SORD	-1.739	NM_003104.3
Thiamin pyrophosphokinase 1	TPK1	-1.744	NM_001042482.1
Nucleoside triphospatase C1orf57	C1orf57	-1.754	NM_032324.1
Sorbitor dehydrogenase	SORD	-1.764	NM_003104.3
	LOC647000	-1.766	XM_929980.2
Adaptor molecule crk	CRK	-1.803	NM_016823.2
Thioredoxin domain containing 12	TXNDC12	-1.820	NM_015913.2
Leucine rich repeat interacting protein 2	LRRFIP2	-1.821	NM_017724.1
Regulation of nuclear pre-mRNA domain containing 1A	RPRD1A	-1.826	NM_018170.3
	LOC92755	-1.832	XR_018705.2
Insulin like growth factor binding protein 5	IGFBP5	-1.853	NM_000599.2
Potassium voltage-gated channel subfamily G member 1	KCNG1	-1.853	NM_002237.3
Proteasome subunit beta type-7	PSMB7	-1.946	NM_002799.2
Tubulin beta chain	TUBB	-1.951	NM_178014.2
CKLF-like MARVEL transmembrane domain containing 6	CMTM6	-1.958	NM_017801.2
Regulation of nuclear pre-mRNA domain-containing protein 1A	P15RS	-1.976	NM_018170.2
Neuronal pentraxin 1	NPTX1	-1.976	NM_002522.2
Ubiquitin-like domain-containing CTD phosphatase 1	UBLCP1	-1.988	NM_145049.1
Tubulin beta chain	TUBB	-2.007	NM_178014.2
	LOC92755	-2.017	XR_016140.2
Elongation of very long chain fatty acids protein 1	ELOVL1	-2.023	NM_022821.2
Basic leucine zipper and W2 domain 2	BZW2	-2.053	NM_014038.1
Acetyl coenzyme A acyltransferase	ACAA2	-2.087	NM_006111.1
	LOC647000	-2.105	XM_929980.2
Myosin X	MYO10	-2.105	NM_012334.1
Ubiquitin-conjugating enzyme E2 J1	UBE2J1	-2.187	NM_016021.2
Solute carrier family 7	SLC7A2	-2.207	NM_001008539.2
	LOC653381	-2.452	XR_017364.1
Fas-associated factor 1	FAF1	-6.342	NM_007051.2
Fas-associated factor 1	FAF1	-8.335	NM_007051.2

Supplementary Fig 1.

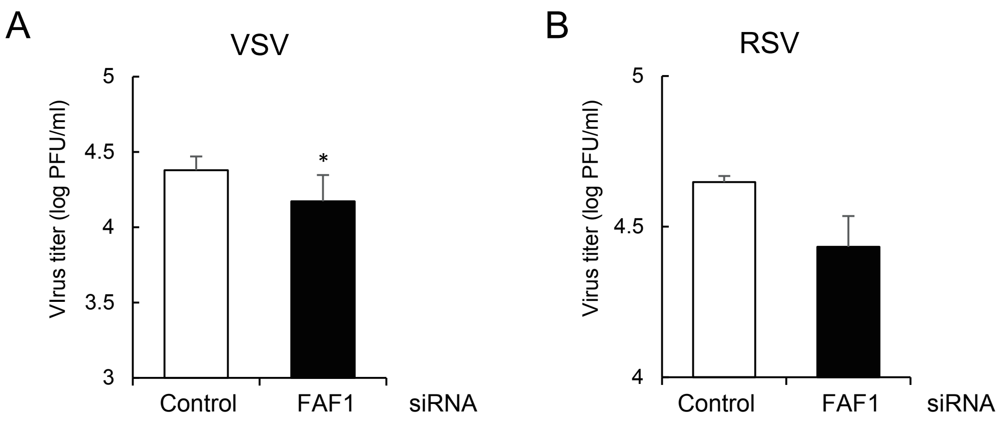

Supplementary Fig 1. Plaque formation assay using VSV and RSV. (A) BHK-21 cells were transfected with control or FAF1 siRNA #2 for 40 h. When cells reached 90% confluence, the medium was removed and cells were incubated with VSV-GFP (100 pfu/well) or uninfected. At 1 h post infection, the culture supernatant was removed and cells were overlaid with 1:1 mixture of 10% MEM and 1% agarose. At 5 days post infection, cells were fixed with 4% paraformaldehyde for 1 h and stained with 0.2 % crystal violet. Plaques were counted, averaged and multiplied by the dilution factor. The experiments were conducted in quadruple. *$p<0.05$ for si-Con vs si-FAF1. (B) HEp-2 cells were transfected with control and FAF1 siRNA #2 for 50 h and cells were incubated with RSV inoculums (50 pfu/well) for 2 h with rocking every 30 min or uninfected. The culture supernatant was removed and cells were overlaid with 1:1 mixture of 10% MEM and 1% agarose. At 5 days post infection, plaques were counted after staining with neutral red and multiplied by the dilution factor. The experiments were conducted in duplicate.
